# The London Hospital's Nurses

**Published:** 1918-10-19

**Authors:** 


*To the Editor of* The Hospital.

### THE LONDON HOSPITAL'S NURSES.

*To the Editor of* The Hospital.

Sir,—To those in the profession the correspondence under the above heading would give the impression that those responsible for the probationers' training at the "London" are too narrow-minded to move with the times, or callous to the true interest of their probationers. It is recognised in the profession that a three years' training is essential for the better appointments—that is to say, three consecutive years in the wards of a recognised training-school. Granted the "London" is the largest hospital, still its nurses can only do a limited share of daily work, and the least intelligent can obviously see that the larger the institution the more there is to learn, and the longer time should be given to get the round in. If it is curtailed some work must be missed out entirely, or else little time spent on it. Owing to lack of time, one gathers, a general training in all branches is not given.

Again, one realises it would be less strenuous for a probationer to have three years to learn practical and theoretical work in, and the patients would benefit as well as the nurse: such an arrangement would also be less tiring for those who teach.

At the end of training no one could state, when the nurse applied for the first appointment, "You are ineligible, as you lack three consecutive years' training in the wards."

If a nurse can be trained in two years, what time is curtailed for the students? If none, why is such a fallacy raised for the nursing staff?

Truly a three years' training costs more, but it is to be hoped the day is not far distant when would-be nurses will pass by the schools that are obviously not giving their best. One hopes the day is near at hand when the "London" will lead the way with a training-school second to none. At present it has a long way to go to reach the goal.

As to salary, the majority of nurses, however keen, have their living to make, sometimes others to help, and a future to provide for. If a hospital can't be supported by its subscriptions, etc., don't let it be at the expense of its women workers—the labourer, as of old, is worthy of his hire.

Shorter hours and a weekly day off would be for the good of nurses and patients. As to "London" nurses receiving a pension, the percentage, methinks, is a very small one. The hours for nurses are long, the demands, mentally and physically, great, the pay small.

The openings for women workers were never so great. There must be a speedy reorganisation in the nursing world if the right women are to be the nurses of the future.

Interested.

